# 

**Published:** 1910-07

**Authors:** 


### CONTENTS.

Hydro-Therapy. By L. Amster, M. D., Atlanta, Ga. .......... 167
Some Thoughts on Tuberculosis. By George Brown, M. D., Atlanta, Ga. .......... 174
Is there too much Surgery? By Dr. W. W. Mangum, A. M., M. D., Rome, Ga. .......... 179
A Clinical Lecture Before the Washington Medical Society. Aphasia. By Tom A. Williams, M. D., Cm., Washington, D. C. .......... 186
Tics in Children and their Treatment By Dr. Henry C. Whelchel, Douglas, Ga. .......... 196
An Unusual Instance of Poisoning with Argyrol injected into the Deep Urethra and Bladder., By Edgar G. Ballinger, M. D., Atlanta, Ga. .......... 201
COMMUNICATIONS .......... 203
EDITORIALS .......... 206
SELECTIONS .......... 209
NEWS AND NOTES .......... 212
BOOK REVIEWS .......... 215
MEDICAL ITEMS .......... 219

